# Prediction of serum neuritin and neuron-specific enolase for prognosis in patients with traumatic brain injury combined with spinal cord injury

**DOI:** 10.5937/jomb0-45469

**Published:** 2024-11-16

**Authors:** Bingbing Pu, Yu Chen, Qingguo Bi, Jian Shen, Lihui Wang, Ye Han

**Affiliations:** 1 Pudong Hospital, Department of Rehabilitation, Shanghai, China; 2 Zhoupu Hospital, Department of Critical Care, Shanghai, China; 3 Pudong Hospital, Department of Medical Equipment, Shanghai, China; 4 People's Hospital of Xuyi County, Department of Pathology, Huaian, Jiangsu Province, China

**Keywords:** serum neuritin, neuron-specific enolase, traumatic brain injury, spinal cord injury, prognostic prediction, serumski neuritin, neuron-specifična enolaza, traumatska povreda mozga, povreda kičmene moždine, prediktivna prognoza

## Abstract

**Background:**

Serum neuritin and neuron-specific enolase (NSE) have predictive value for the prognosis of patients with combined traumatic brain injury (TBI) and spinal cord injury (SCI). Studying their predictive effects has positive value for disease control and treatment.

**Methods:**

Sixty patients with combined TBI and SCI were recruited and rolled into three groups according to prognosis: Group I (n=42, favourable prognosis), Group II (n=11, poor prognosis), and Group III (n=7, death). Clinical indicators were compared between the groups, and the predictive value of different indicators for prognosis was analyzed.

**Results:**

The proportion of patients with combined injuries to other organs and hypotension, as well as levels of platelets (PLT), D-dimer (D-D), antithrombin III (AT-III), S100 protein (S100), NSE, and serum neurofilament levels were significantly higher in Groups II and III compared to Group I. Conversely, the Glasgow Coma Scale (GCS) scores were significantly lower in Group I (P<0.05). Multivariable logistic regression analysis revealed that other organ injuries, GCS score, PLT, D-D, and AT-III significantly influenced the prognosis of TBI combined with SCI patients (P<0.05), while hypotension, NSE, serum neurofilament levels, S100, and accompanying organ injuries were highly correlated with the prognosis of TBI combined with SCI patients (P<0.001). The predictive sensitivity, specificity, accuracy, positive predictive value, and negative predictive value of NSE combined with serum neurofilament in predicting the prognosis of TBI combined with SCI patients were significantly higher than the singular predictive efficacy of NSE or serum neurofilament alone (P<0.05).

**Conclusions:**

To evaluate the prognosis of TBI combined with SCI patients, consideration should be given to factors such as other organ injuries, hypotension, consciousness assessment, and levels of various biomarkers. Furthermore, combined testing of serum neurofilament and NSE can more accurately predict the prognosis of TBI combined with SCI patients.

## Abstract (sr)

**Uvod:**

Serumski neuritin i neuron-specifična enolaza (NSE) imaju prediktivnu vrednost za prognozu kod pacijenata sa traumatskom povredom mozga (TBI) u kombinaciji sa povredom kičmene moždine (SCI). Proučavanje njihovih prediktivnih efekata ima pozitivnu vrednost za kontrolu bolesti i lečenje.

**Metode:**

Odabrano je šezdeset pacijenata sa kombinacijom TBI i SCI koji su podeljeni u tri grupe prema prognozi: Grupa I (n=42, povoljna prognoza), Grupa II (n=11, loša prognoza) i Grupa III (n=7, smrtni ishod). Upoređivani su klinički pokazatelji između grupa i analizirana je prediktivna vrednost različitih pokazatelja u smislu prognoze.

**Rezultati:**

U grupama II i III, u poređenju sa grupom I, uočena je značajno veća proporcija pacijenata sa kombinovanim povredama drugih organa i hipotenzijom, kao i veći nivoi trombocita (PLT), D-dimera (D-D), antitrombina III (ATIII), proteina S100 (S100 ), NSE i serumskih neurofilamenta. Suprotno tome, rezultati Glazgovske skale kome (GCS) su bili značajno niži u Grupi I (P<0,05). Multivarijantna logistička regresiona analiza je pokazala da druge povrede organa, rezultati GCS-a, PLT, D-D i AT-III značajno utiču na prognozu kod pacijenata sa TBI u kombinaciji sa SCI (P<0,05), dok su hipotenzija, NSE, nivoi serumnih neurofilamenta, S100 i prateće povrede organa bile u značajnoj korelaciji sa prognozom kod pacijenata sa TBI u kombinaciji sa SCI (P<0,001). Prediktivna osetljivost, specifičnost, tačnost, pozitivna prediktivna vrednost i negativna prediktivna vrednost NSE u kombinaciji sa serumskim neurofilamentom u predviđanju prognoze kod pacijenata sa TBI kombinovanim sa SCI bile su značajno veće nego pojedinačna prediktivna efikasnost NSE ili serumskog neurofilamenta (P<0,05).

**Zaključak:**

Za procenu prognoze kod pacijenata sa TBI u kombinaciji sa SCI, treba uzeti u obzir faktore kao što su druge povrede organa, hipotenzija, procena svesti i nivoi različitih biomarkera. Takođe, kombinovano testiranje serumskog neurofilamenta i NSE može preciznije predvideti prognozu kod pacijenata sa TBI u kombinaciji sa SCI.

## Introduction

Traumatic brain injury (TBI) and spinal cord injury (SCI) are currently among the most common causes of death and disability worldwide. TBI refers to brain tissue damage caused by external forces, which usually leads to neurological dysfunction, manifested as symptoms such as headache, consciousness disorders, and abnormal pupils [1] [2]. SCI is usually due to external force on the back or spinal cord, resulting in neurological damage, leading to symptoms such as limb paralysis and urinary disorders [3] [4]. Currently, the treatment of TBI and SCI mainly includes early maintenance of life functions and surgical treatment [5] [6]. Patients with combined TBI and SCI usually have more complex conditions and are more difficult to treat [7] [8].

Serum neuritin and neuron-specific enolase (NSE) are currently hot research topics, and they are widely believed to play an imperative role in the recovery process of nerve cell injury. Studies have noted that serum neuritin can stimulate neuronal growth and regeneration and promote dopamine production [9]. NSE is mainly involved in the metabolic processes and growth and infiltration of neurons [10] [11]. The levels of serum neuritin and NSE are closely related to the growth and regeneration of nerve cells and may affect the prognosis of patients with combined cranial and SCIs [12]. For patients with combined cranial and SCIs, changes in the levels of serum neuritin and NSE can be utilized as predictors of neural system injury after cranial and SCI [13] [14].

TBI and SCI are major contributors to severe disability and mortality. Accurate prognosis assessment and effective treatment have been longstanding challenges in the medical field. This study aimed to investigate the predictive value of serum neurofilament and NSE in the prognosis of patients with TBI combined with SCI and explore their potential clinical implications. Through monitoring serum NSE levels combined with NSE measurements and clinical observations of patients receiving integrated treatment for cranial and spinal injuries, we aimed to provide new insights into the prevention and treatment of TBI combined with SCI, thereby offering more precise diagnostic and therapeutic approaches in clinical practice. The findings of this study are expected to provide important clinical guidance and references for improving the prognosis and quality of life of patients with TBI combined with SCI.

## Materials and methods

### Research design

A prospective cohort study design was employed to collect data from patients with TBI combined with SCI. Long-term follow-up observations were conducted to assess the role of serum neurofilament and NSE in predicting patient outcomes.

### Research object

In this study, 60 patients with TBI combined with SCI admitted to our Hospital from June 2022 to December 2023 were selected as the research subjects. Among them, there were 32 male patients and 28 female patients, with ages ranging from 20 to 69 years (mean age of (47.28±9.09) years), body mass index (BMI) ranging from 19.21 to 24.09 kg/m^2^ (mean BMI of (22.33±42.12) kg/m^2^), and educational years ranging from 8 to 17 years (mean educational years of (12.42±1.09) years). Based on the prognosis of the patients, the 60 TBI combined with SCI patients were divided into three groups: Group I (favourable prognosis) included 42 cases, with 22 males and 20 females, average age of (46.45±8.28) years, average BMI of (22.33±2.34) kg/m^2^, and average educational years of (12.33±1.22) years; Group II (poor prognosis) included 11 cases, with 6 males and 5 females, average age of (48.23±8.46) years, average BMI of (22.56±2.23) kg/m^2^, and average educational years of (12.38±1.37) years; Group III (deceased) included 7 cases, with 4 males and 3 females, average age of (47.16±8.28) years, average BMI of (22.12±2.73) kg/m^2^, and average educational years of (12.56±1.29) years. There were no significant differences in gender, age, BMI, and educational years among the three groups (P>0.05), suggesting comparability among the study groups. This study was approved by the Pudong Hospital, Shanghai 201399, China Medical Ethics Committee. The inclusion and exclusion criteria for the study subjects were outlined as follows:

Inclusion criteria: (1) Complete medical records; (2) Age over 18 years; (3) No other brain diseases; (4) Consciousness clear and highly cooperative; (5) Patient and family members signed informed consent.

Exclusion criteria: (1) Presence of other imperative organ diseases; (2) Presence of malignant tumours; (3) Presence of genetic diseases; (4) Communication barriers; (5) Unwillingness to participate in this study.

### Research methodologies

The Glasgow Coma Scale (GCS) was employed to classify TBI severity. Patients were graded as having mild injury with a GCS score of 13–15, moderate injury with a GCS score of 9–12, or severe injury with a GCS score of 3–8. The Glasgow Outcome Scale (GOS) was utilized to evaluate the prognosis of TBI patients. Patients who died after treatment were assigned a score of 1; those who survived in a vegetative state were assigned a score of 2; those who were conscious but had a severe disability and required assistance with daily activities were assigned a score of 3; those with a moderate disability who were able to live independently were assigned a score of 4, and those with no apparent disability but with mild mental impairment were assigned a score of 5. Relevant indicators were measured in the three patient groups.

### General data collection

Data were collected and organized for each group of patients, including the number of patients with organ injuries, the number of patients with hypotension, GCS scores, heart rate (HR), respiratory rate (RR), and other clinical data.

### Platelet count (PLT) testing

PLT was measured using an automated haematology analyzer (BC-6600 model, Guangdong Shenzhen, Jisheng (Shanghai) Medical Equipment Co., Ltd., registration number 20172401464). Two millilitres of venous blood were collected from the patient, and the instrument-specific parameters were set on the analyzer. The blood sample was then sent through a cleaning tube into the analyzer, which automatically extracted the blood sample and installed the slide. During the automatic analysis process, the analyzer separated the platelets from other blood cells by performing automatic liquid addition, mixing, shaking, and deaeration, and automatically calculated and recorded the platelet count per litre of blood.

### Detection of serum D-dimer (D-D), serum antithrombin III (AT-III), serum S100 protein, NSE, and serum neurofilament

Enzyme-linked immunosorbent assay was utilized to detect D-D, AT-III, S100β, NSE, and Neuritin. The kits were purchased from Shanghai Senxiong Biotechnology Co., Ltd. The biological samples, such as serum or plasma, were centrifuged to remove impurities, diluted, and added to each well of the plate along with an appropriate amount of antibodies to achieve specific capture and binding through synergistic action. A washing buffer was utilized to remove impurities from the plate wells and reduce the influence of nonspecific adsorption and background noise. The pre-processed samples were applied to each well to bind with the capture antibodies and labelled antibodies. Then, secondary antibodies with fluorescence or enzyme labelling were applied to form a complex. A washing buffer was utilized again to remove impurities and reduce nonspecific adsorption and background noise. If the labelled antibody was enzyme-labelled, then an enzyme substrate was applied in the final step to form an enzyme-substrate complex. A colour substrate was then applied to induce a colour reaction. This step mainly integrated the reagents into the complex, which was associated with the enzyme-labelled antibody module. The quantitative analysis of D-D, AT-III, S100β, NSE, and Neuritin in the samples was performed using equipment such as an enzyme-labelled instrument (BJKA-01, Beijing Kaiao Technology Development Co., Ltd.) to read signals such as luminescence and absorbance.

### Observation indexes

1. Univariate analysis was conducted to assess the impact of organ injuries, hypotension incidence, GCS score, HR, RR, PLT, D-D, AT-III, serum S100β protein, NSE, and serum neurofilament levels on patients with TBI combined with SCI. This analysis aimed to identify prognostic factors affecting these patients.

2. A multiple logistic regression analysis was performed to investigate the prognostic factors of patients with TBI and SCI.

3. A comparative analysis of the predictive effects of various detection methodologies on the prognosis of patients with TBI and SCI was carried out. The indicators analyzed included sensitivity (*Sen*), specificity (*Spe*), accuracy (*Acc*), positive predictive value, and negative predictive value, with the GOS score serving as the diagnostic gold standard. The Sen was calculated using Eq. (1), *Spe *using Eq. (2), Acc using Eq. (3), positive predictive value using Eq. (4), and negative predictive value using Eq. (5). In these equations, DM represents the actual number of patients with poor prognosis as determined by the three detection methods, *PM *represents the number of patients diagnosed as having a poor prognosis by the GOS score, *DB *represents the actual number of patients with favourable prognosis as determined by the three detection methods, *PB *represents the number of patients diagnosed as having a favourable prognosis by the GOS score, *YDM* represents the number of patients determined by the three detection methods to have a poor prognosis, and *YDB *represents the number of patients determined by the three detection methods to have a favourable prognosis.


(1) Sensitivity =\frac{DM}{PM}\times 100 \% (2)

(2) Specificity =\frac{DB}{PB}\times 100 \% (3)

(3) Accuracy =\frac{DM+DB}{PM+PB}\times 100 \% (4)

(4) Positive \ predictive \ value =\frac{DM}{YDM}\times 100 \% (5)

(5) Negative \ predictive \ value =\frac{DB}{YDB}\times 100 \% (6)

### Statistical methodologies

Data recording and summarization were performed using Excel 2016, while data statistics and analysis were conducted using SPSS 20.0. For continuous data, mean values ± standard deviations (x̄±S) were utilized and analyzed using t-tests. Percentages (%) were utilized to represent count data and analyzed using the chi-square test. *P*<0.05 indicated statistical significance. A significance level of *P*<0.001 was used to indicate a highly significant difference.

## Results

### Univariate analysis of prognostic factors in patients with TBI combine with SCI

### Comparison of patients with combined organ injuries

**Figure 1:**
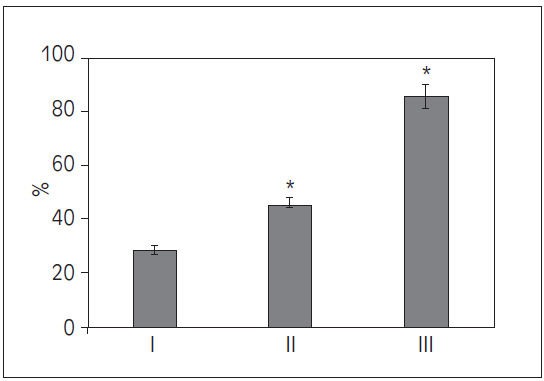
Comparison of proportions of patients with combined organ injuries among different prognostic groups (*P<0.05 vs. Group I).

After data organization, it was found that in this study, Group I had 12 cases of patients with combined organ injuries, accounting for 28.57%; Group II had 5 cases, accounting for 45.45%; and Group III had 6 cases, accounting for 85.71%. Upon comparison, the proportion of patients with combined organ injuries in Groups II and III was significantly higher than that in Group I (*P*<0.05) (Figure 1).

### Comparison of patients with hypotension

**Figure 2:**
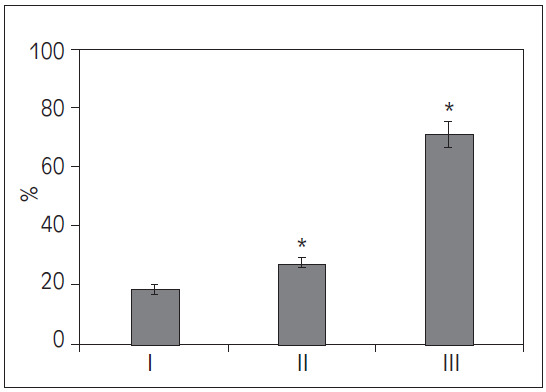
Comparison of proportions of patients with hypotension among different groups (*P<0.05 vs. Group I).

Upon data organization, it was found that in this study, there were 8 cases of patients with hypotension in Group I, accounting for 19.05%; in Group II, there were 3 cases, accounting for 27.27%; and in Group III, there were 5 cases, accounting for 71.43%. Upon comparison, the proportion of patients with hypotension was significantly higher in Groups II and III than in Group I (*P*<0.05) (Figure 2).

### Comparison of GCS scores

**Figure 3:**
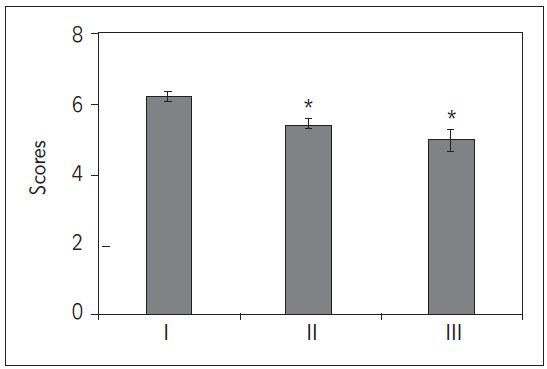
Comparison and analysis of GCS scores of patients with different prognoses of TBI combined with SCI. (*P<0.05 vs. group I).

Upon data organization, it was found that in this study, the GCS score for patients with TBI combined with SCI was (5.98±1.83) in Group I, (5.27±1.99) in Group II, and (4.97±1.27) in Group III. Upon comparison, the GCS scores in Groups II and III were significantly lower than those in Group I (*P*<0.05) (Figure 3).

### Basic data comparison

**Figure 4:**
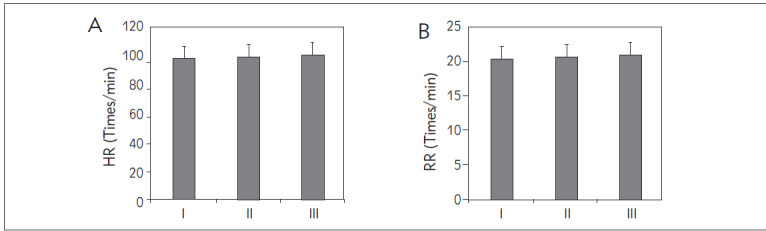
Comparison of HR and RR among the three groups.

Upon data organization, it was observed that in this study, the HR of patients in Group I was (97.35± 10.21) beats/min, with a RR of (18.85±2.33) breaths/min; in Group II, the HR was (98.62±9.67) beats/min, with an RR of (18.92±2.98) breaths/min; and in Group III, the HR was (99.87±11.78) beats/min, with an RR of (19.76±2.09) breaths/min. Upon comparison, no significant statistical differences in HR and RR were found among the three groups (*P*>0.05) (Figure 4).

### Laboratory indicator comparison

**Figure 5:**
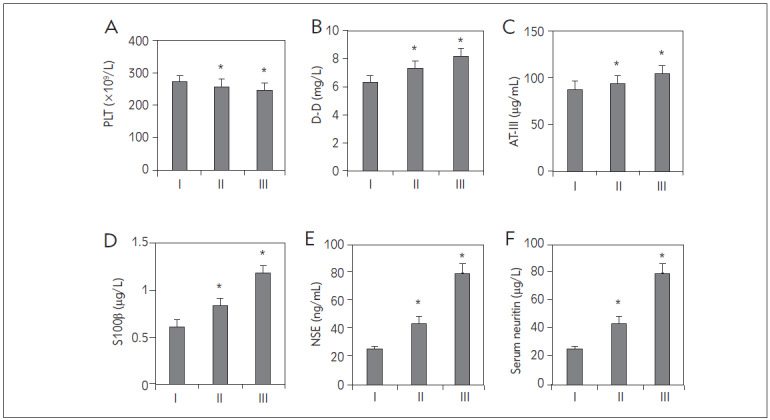
Comparison of laboratory indicator levels among the three groups (A: PLT, B: D-D, C: AT-III, D: S100β, E: NSE, F: serum neurofilament *P<0.05 vs. Group I).

Upon data organization, it was found that in this study, patients in Group I had a PLT of (269.73±12.39) × 10^9^/L, D-D level of (6.15±1.03) mg/L, AT-III level of (86.73±9.37) μg/mL, S100β protein level of (0.61±0.23) μg/L, NSE level of (24.76±5.38) ng/mL, and serum neurofilament level of (76.25±8.09) μg/L; patients in Group II had a PLT of (258.46±10.18) × 10^9^/L, D-D level of (7.26±1.28) mg/L, AT-III level of (93.11±10.93) μg/mL, S100β protein level of (0.83±0.20) μg/L, NSE level of (43.82±3.94) ng/mL, and serum neurofilament level of (94.72±8.10) μg/L; patients in Group III had a PLT of (247.33±10.29) × 10^9^/L, D-D level of (7.92±1.39) mg/L, AT-III level of (102.79±12.18) μg/mL, S100β protein level of (1.17±0.32) μg/L, NSE level of (78.26±5.43) ng/mL, and serum neurofilament level of (103.78±10.83) μg/L. Upon comparison, patients in Groups II and III exhibited significantly higher levels of PLT, D-D, AT-III, S100β protein, NSE, and serum neurofilament compared to Group I (*P*<0.05) (Figure 5).

### Multivariate logistic regression analysis of prognostic factors in patients with TBI combine with SCI

Table 1 presents the results of multivariable logistic regression analysis on factors influencing the prognosis of patients with TBI combined with SCI. The analysis indicates that other organ injuries, hypotension, GCS score, PLT, D-D, AT-III, NSE, serum neurofilament, and S100β protein significantly impact the prognosis of TBI combined with SCI patients (*P*<0.05). Specifically, hypotension, NSE, serum neurofilament, S100β protein, and accompanying organ injuries are highly correlated with the prognosis of TBI combined with SCI patients (*P*<0.001).

**Table 1:** Multivariate Logistic regression analysis of prognostic factors in patients with TBI complicated with SCI.

Factor	β	SE	Wald x^2^	OR	*P*
Merge	1.134	0.458	4.737	2.631	<0.001
Low	2.716	0.415	8.369	2.883	<0.001
GCS	1.933	0.367	5.535	2.436	0.012
PLT	0.843	0.455	4.934	1.643	0.003
D-D	0.783	0.483	4.257	1.342	0.041
AT-III	1.827	0.592	5.853	2.264	0.022
S100b	2.357	0.416	4.633	2.093	<0.001
NSE	2.715	0.433	5.788	3.771	<0.001
Neuritin	2.276	0.519	3.743	3.743	<0.001

### Comparative analysis of NSE and serum neuritin in predicting prognosis of patients with TBI complicated with SCI

**Figure 6:**
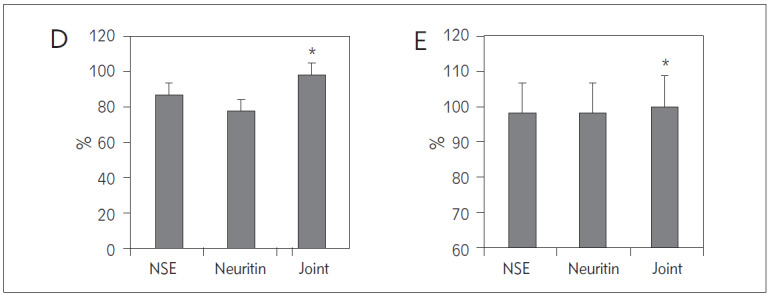
Comparison and analysis of the effects of NSE and serum neuritin in predicting the prognosis of patients with TBI combined with SCI. (A: Sen, B: Spe, C: Acc, D: positive predictive value, E: negative predictive value, *P<0.05 vs. single prediction).

Statistical analysis was conducted to compare and calculate the prognostic predictive effects of serum neurofilament and NSE in patients with TBI combined with SCI. The results showed that NSE had a sensitivity of 94.44%, specificity of 92.86%, accuracy of 93.33%, positive predictive value of 85.00%, and negative predictive value of 97.50% in predicting the prognosis of TBI combined with SCI patients. Serum neurofilament had a sensitivity of 88.89%, specificity of 90.48%, accuracy of 90.00%, positive predictive value of 76.19%, and negative predictive value of 97.44% in predicting the prognosis of TBI combined with SCI patients. When combined, NSE and serum neurofilament exhibited a sensitivity of 100.00%, specificity of 97.62%, accuracy of 98.33%, positive predictive value of 94.74%, and negative predictive value of 100.00% in predicting the prognosis of TBI combined with SCI patients (Figure 6). Comparatively, the combination of NSE with serum neurofilament demonstrated significantly higher sensitivity, specificity, accuracy, positive predictive value, and negative predictive value in predicting the prognosis of TBI combined with SCI patients compared to the singular predictive efficacy of NSE or serum neurofilament alone (*P*<0.05).

## Discussion

TBI combined with SCI is a severe trauma commonly associated with high-intensity sports or motor vehicle accidents [15]. It results in significant neurological impairments such as limb numbness, motor dysfunction, and sensory deficits, often accompanied by complications such as intracranial haemorrhage and brain damage. Prompt and effective treatment and rehabilitation for these patients are crucial. In recent years, serum neurofilament and NSE have shown promise in diagnosis and prognosis prediction. They can aid in rapid diagnosis of brain injury, monitor recovery post-treatment, and predict neurological function recovery and prognosis [3] [16]. Therefore, a comprehensive analysis of these biomarkers is significant for diagnosing and predicting disease progression in patients. Based on this, our study analyzed the predictive efficacy of serum neurofilament and NSE for the prognosis of patients with TBI combined with SCI.

This study found that the proportion of patients with combined organ injuries and hypotension was significantly higher in Groups II and III compared to Group I (*P*<0.05), indicating that the presence of additional organ injuries and hypotension may contribute to increased complexity in patient conditions and worsen prognosis. Literature suggests that patients with multi-organ injuries generally have poorer outcomes, particularly when there is an impairment in cardiopulmonary function, leading to increased mortality and complication rates [17] [18]. Hypotension may be a common complication in patients with TBI combined with SCI and is closely associated with poor prognosis [19]. Hypotension can result in inadequate cerebral perfusion, exacerbating brain injury, and also affecting the function of other organs, worsening overall systemic condition. Studies indicate that early and effective blood pressure control is crucial for improving patient outcomes [20]. The GCS score is a common indicator used to assess consciousness levels, with a low GCS score indicating severe neurological impairment and closely correlating with poor prognosis [21] [22]. This study concluded that GCS scores in Groups II and III were significantly lower than those in Group I (*P*<0.05), suggesting that lower GCS scores are associated with worse patient outcomes. Additionally, the levels of PLT, D-D, AT-III, S100β, NSE, and serum neurofilament were significantly higher in Groups II and III compared to Group I (*P*<0.05), suggesting a general elevation of these biomarkers in patients with poor prognosis. PLTs, D-D, AT-III, S100β, NSE, and serum neurofilament are typically released or increased in serum levels in response to tissue injury or inflammatory stimuli [23] [24] [25]. These biomarkers exhibit varying changes under different types of injury or disease states, but they generally reflect the body’s stress response and extent of damage [26] [27].

Further analysis through multivariable logistic regression revealed a strong correlation between hypotension, NSE, serum neurofilament, S100β, combined organ injuries, and the prognosis of TBI combined with SCI (*P*<0.001). This indicates that these factors are important determinants affecting the prognosis of patients with TBI combined with SCI, and their presence or level changes may closely relate to prognosis outcomes. Notably, NSE is a neuron specific enzyme primarily found in the nervous system. When neurons are damaged, NSE is released into the bloodstream, making its serum levels useful for assessing the extent of neurological injury. Serum neurofilament is a major component of neuronal cell membranes, and elevated levels may reflect neuronal membrane damage or injury. Based on this, the study investigated the predictive efficacy of serum neurofilament and NSE for TBI combined with SCI, revealing that the combined measurement of NSE with serum neurofilament significantly outperformed individual biomarkers in sensitivity, specificity, accuracy, positive predictive value, and negative predictive value (*P*<0.05). This suggests that combining these two biomarkers can more accurately predict the prognosis of patients with TBI combined with SCI. These findings are clinically significant, highlighting the importance of integrating multiple biomarker information when assessing the prognosis of patients with TBI combined with SCI, which can enhance the accuracy of prognosis assessment and provide a more reliable basis for patient treatment and management.

## Conclusion

In summary, this study analyzed the baseline data and laboratory indicator levels of patients with TBI combined with SCI under different prognostic outcomes, comparing the predictive efficacy of serum neurofilament and NSE for the prognosis of TBI combined with SCI. The study findings suggested that when assessing the prognosis of TBI combined with SCI patients, it is important to consider factors such as other organ injuries, hypotension, consciousness assessment, and levels of various biomarkers. Furthermore, the combined detection of serum neurofilament and NSE can more accurately predict the prognosis of TBI combined with SCI patients, demonstrating high clinical utility and positive significance. Therefore, these findings can be applied in clinical practice with substantial guidance value. However, a limitation of this study is the small sample size, necessitating further research and validation.

## Dodatak

### Funding

The research is supported by the Project of Key Medical Specialty and Treatment Center of Pudong Hospital of Fudan University (No. Tszb2023-10).

### Conflict of interest statement

All the authors declare that they have no conflict of interest in this work.
